# Acute Esophageal Necrosis in a Septic Patient with a History of Cardiovascular Disease

**DOI:** 10.1155/2020/1416743

**Published:** 2020-05-11

**Authors:** Michael Coles, Victoria Madray, Pearl Uy

**Affiliations:** Augusta University, 1120 15 Street, Augusta, Georgia 30912, USA

## Abstract

Acute esophageal necrosis (AEN), or colloquially named “black esophagus,” is a rare clinical condition often associated with ischemic injury to the esophagus secondary to splanchnic vasoconstriction during hypotensive episodes. We present a case of a 78-year-old man with extensive cardiovascular disease who was initially admitted for gallstone pancreatitis and possible cholangitis. His hospital course was complicated by possible sepsis secondary to aspiration pneumonia and hematemesis secondary to acute ischemic esophageal necrosis as noted on upper endoscopy. Interestingly, the patient only had a transient episode of hypotension (approximately 35 minutes) not requiring vasopressor support, which improved with fluid resuscitation, and endoscopic retrograde cholangiopancreatography (ERCP) done 3 days prior showed normal esophageal mucosa. Clinicians should be aware of the possibility of acute esophageal necrosis as a potential etiology of gastrointestinal (GI) bleed in patients with cardiovascular disease and sepsis.

## 1. Case Report

A 78-year-old Caucasian male with past medical history significant for coronary artery disease with multivessel coronary artery bypass grafting, paroxysmal atrial fibrillation on apixaban therapy, heart failure with reduced ejection fraction (∼37%), chronic kidney disease stage III, and chronic abdominal aortic aneurysm (AAA) with graft repair in remote past was admitted and treated for gallstone pancreatitis and possible cholangitis. On hospital day 2, he produced 50 mL of coffee ground emesis and experienced a transient episode of hypotension to 70/50 mmHg, which improved with 1 L fluid bolus. The patient was treated empirically for aspiration pneumosepsis given hypotension and equivocal chest X-ray findings, but no organism was identified on culture data. Subsequent magnetic resonance imaging study showed filling defect of the common bile duct without dilation suspicious for cholangitis secondary to stone or sludge. A following endoscopic retrograde cholangiopancreatography (ERCP) showed duodenal diverticulum and erosive duodenitis; a common bile duct (CBD) stent was placed. On hospital day 4, a follow-up ERCP showed normal esophagus and no filling defect in the CBD, with persistent duodenitis. However, on hospital day 7, the patient complained of acute chest pain accompanied by 200 mL of bright red emesis. Of note, apixaban (5 mg twice daily) was restarted the night before, and INR was elevated at 2.5. The patient remained hemodynamically stable with a hemoglobin and hematocrit of 9.1 mg/dL and 27.4%, respectively, suggestive of hemostasis. However, his white blood cell count increased dramatically from 11,000 to 18,000/mm.^3^

**Figure 1:**
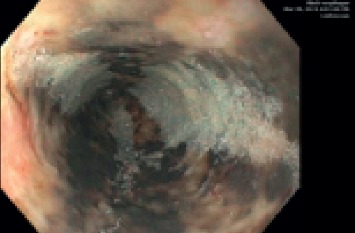
Diffuse black discoloration of the esophagus with linear ulcerated mucosa.

**Figure 2:**
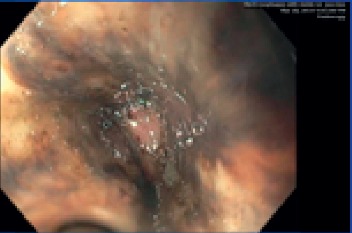
Circumferential black discoloration of the distal esophageal mucosa with clear demarcation with the uninvolved GE junction.

Subsequent esophagogastroduodenoscopy (EGD) revealed circumferential black esophagus extending entirely from the proximal to distal esophagus (gastroesophageal (GE) junction noted at 40 cm from the incisors) and old blood in the gastric cavity, but no signs of active bleeding (Figures 1 and 2).

The images seen on endoscopy showed diffuse black discoloration, linear ulcers, and stark demarcation of involved and uninvolved mucosa at the gastroesophageal junction in the absence of toxin ingestion. A diagnosis of acute esophageal necrosis (AEN) was made, and the patient was continued on intravenous proton pump inhibitors (PPI), antibiotics, and fluid hydration; apixaban was held. The rest of his hospitalization was unremarkable, and he was discharged to a long-term care facility. We intend to perform repeat endoscopy to confirm resolution of mucosal injury and rule out stricture formation.

## 2. Discussion

Acute esophageal necrosis (AEN) is rare with less than one hundred patient cases published in the literature and a prevalence of 0.2% [1]. Typically, AEN presents in critically ill patients with hemodynamic compromise such as cardiovascular disease or sepsis requiring intensive inotropic support [2]. The diagnosis of AEN can be made with endoscopic evidence of necrotic esophageal mucosa in the above-described clinical setting. Histologic evidence can confirm AEN but is unnecessary to make a diagnosis [3]. Our patient's hemodynamic stability despite panesophageal necrosis remains nebulous. Alternative diagnoses (pseudomenlanosis, acanthosis nigricans, and malignant melanoma) are possible and no tissue biopsy confirmed necrosis; however, the patient's symptom constellation and acuity of presentation were more consistent with AEN.

The etiology of AEN is multifactorial and largely thought to be due to an ischemic insult leading to decreased esophageal perfusion and impairment of the mucosal barrier of the esophagus. During episodes of hypotension or shock, the body's protective mechanism of splanchnic vasoconstriction results in decreased perfusion of visceral organs such as the esophagus, stomach, and intestines with loss of >90% of gastric blood flow [4]. The distal esophagus is most often involved in AEN, likely due to its decreased vascular supply in the face of a global hypoperfused state and vasoconstriction. A “two-hit” hypothesis theory has developed suggesting hypoperfusion increases opportunity for injury to the mucosa by a second entity such as toxins or acids that results in rapid onset of necrosis unless addressed and treated [1]. Therefore, a high level of suspicion for acute esophageal necrosis as a differential diagnosis for gastrointestinal bleed is imperative among patients with cardiovascular disease and sepsis given its high mortality rate of 32% and potential rapidity of onset requiring urgent treatment [1].

A notable feature in our case was the rapid necrosis secondary to one brief period of hypotension that hemodynamically responded to fluid administration. We propose that the extensive comorbid vascular disease present in our patient, especially the previous surgical treatment of AAA, resulted in ischemic necrosis of the distal esophagus consequent to limited hypotensive stress. The distal esophagus is normally perfused via the esophageal branch of the left gastric artery arising from the celiac trunk [1]. Although computed tomography angiography was not performed in our patient, esophageal vasculature supply proximal to the celiac trunk was also likely not well collateralized at baseline given the patient's vasculopathic manifestations elsewhere (coronary artery disease and chronic kidney disease). Already tenuous tissue perfusion was further compromised by a brief period of hypotension in the midst of sepsis possibly explaining panesophageal involvement.

AEN treatment aims to restore hemodynamic stability in hypotensive patients using fluids, blood transfusion, and PPI therapy [4, 5]. Patients should be nil-per-os due to risk of perforation of necrotic tissue with passage of food or tubing [4, 5]. After stabilization and treatment, repeat endoscopy can be utilized to confirm resolution of mucosal injury and rule out complication of AEN such as stricture formation, which occurs in up to 25% of these cases [5].

AEN is a life-threatening cause of patients presenting with bloody emesis. Hence, expeditious workup including endoscopy is recommended in critically ill patients presenting with hematemesis to define the source of bleeding. Although rare, AEN should be considered in the differential diagnosis, particularly in patients with coexisting extensive vascular disease or a history of septic shock. Since the genesis of AEN results from a critical reduction in esophageal blood flow, this entity may be reversible with supportive management that includes prompt attention to the cardiovascular system that optimizes splanchnic blood flow.
